# Silver, Gold, and Iron Oxide Nanoparticles Alter miRNA Expression but Do Not Affect DNA Methylation in HepG2 Cells

**DOI:** 10.3390/ma12071038

**Published:** 2019-03-29

**Authors:** Kamil Brzóska, Iwona Grądzka, Marcin Kruszewski

**Affiliations:** 1 Institute of Nuclear Chemistry and Technology, Centre for Radiobiology and Biological Dosimetry, Dorodna 16, 03-195 Warsaw, Poland; i.gradzka@ichtj.waw.pl (I.G.); m.kruszewski@ichtj.waw.pl (M.K.); 2 University of Information Technology and Management, Faculty of Medicine, Department of Medical Biology and Translational Research, Sucharskiego 2, 35-225 Rzeszów, Poland; 3 Institute of Rural Health, Department of Molecular Biology and Translational Research, Jaczewskiego 2, 20-090 Lublin, Poland

**Keywords:** silver nanoparticles, gold nanoparticles, superparamagnetic iron oxide nanoparticles, miRNA expression, DNA methylation, epigenetics

## Abstract

The increasing use of nanoparticles (NPs) in various applications entails the need for reliable assessment of their potential toxicity for humans. Originally, studies concerning the toxicity of NPs focused on cytotoxic and genotoxic effects, but more recently, attention has been paid to epigenetic changes induced by nanoparticles. In the present research, we analysed the DNA methylation status of genes related to inflammation and apoptosis as well as the expression of miRNAs related to these processes in response to silver (AgNPs), gold (AuNPs), and superparamagnetic iron oxide nanoparticles (SPIONs) at low cytotoxic doses in HepG2 cells. There were no significant differences between treated and control cells in the DNA methylation status. We identified nine miRNAs, the expression of which was significantly altered by treatment with nanoparticles. The highest number of changes was induced by AgNPs (six miRNAs), followed by AuNPs (four miRNAs) and SPIONs (two miRNAs). Among others, AgNPs suppressed miR-34a expression, which is of particular interest since it may be responsible for the previously observed AgNPs-mediated HepG2 cells sensitisation to tumour necrosis factor (TNF). Most of the miRNAs affected by NP treatment in the present study have been previously shown to inhibit cell proliferation and tumourigenesis. However, based on the observed changes in miRNA expression we cannot draw definite conclusions regarding the pro- or anti-tumour nature of the NPs under study. Further research is needed to fully elucidate the relation between observed changes in miRNA expression and the effect of NPs observed at the cellular level. The results of the present study support the idea of including epigenetic testing during the toxicological assessment of the biological interaction of nanomaterials.

## 1. Introduction

The intensive development of nanotechnology brings great impacts on industry, medicine and many other aspects of society. An increasing use of nanoparticles (NPs) in various applications entails the need for reliable assessment of their potential toxicity for humans. Initially, studies concerning the toxicity of NPs focused on cytotoxic and genotoxic effects [1]. More recently, attention has been paid to epigenetic changes induced by NPs and epigenetic mechanisms underlying observed cytotoxic effects [2,3]. Epigenetics constitute an important link between genotype and phenotype, and plays a critical role in the regulation of numerous cellular processes, such as DNA replication and gene expression. Epigenetic regulatory mechanisms include, among others, DNA methylation, histone modification, chromatin remodelling, and expression of non-coding RNAs, including miRNAs. Epigenetic modifications can be very stable and passed on to multiple generations, however, it is becoming increasingly recognized that they can also change dynamically in response to environmental stressors and that altered epigenetic mechanisms can play an essential role in the development and progression of cancer and other diseases [4,5].

It has been shown that several types of nanoparticles induce changes in miRNA expression [6,7,8,9,10,11,12,13,14], DNA methylation [15,16,17,18,19,20,21,22,23,24,25,26], as well as histone modifications such as acetylation [20,27], phosphorylation [28,29] and methylation [30]. Three of the most prevalent nanomaterials used in medicine and industry are silver (AgNPs), gold (AuNPs), and superparamagnetic iron oxide nanoparticles (SPIONs). Both AgNPs and AuNPs have been shown to alter miRNA expression and induce DNA methylation [6,7,11,12,17,20,21]. Moreover, it has been shown that both types of nanoparticles alter histone H3 phosphorylation [28,29,31,32], while AgNPs also alter histone H3 methylation [30] and decrease its acetylation [20]. On the other hand, literature on the epigenetic effects of SPIONs is very limited. It has been reported that Fe_2_O_3_ SPIONs affect miRNA expression in NIH/3T3 cells [9] and DNA methylation in MCF-7 cells [33]. Epigenetic effects of Fe_3_O_4_ SPIONs have not been studied so far.

In our previous studies, we have extensively analysed the cytotoxicity of AgNPs in cellular and animal models. Using HepG2 and A549 cell lines as an experimental system, we have shown that cellular response to AgNPs is related to the basal activity of cellular signalling pathways [34] and that HepG2 cells can adapt to AgNPs-induced stress [35]. Recently, we have shown that AgNPs, and to a lesser extent AuNPs and SPIONs, can modify the cellular response to external stimuli, such as tumour necrosis factor [36]. Interestingly, SPIONs showed no toxicity in HepG2 cells and were even able to induce cell proliferation in a clonogenic assay [36].

As we and others have previously observed, cellular response to NPs usually consists of changes in the expression of genes related to inflammation and apoptosis [34,35,36,37,38]. To further investigate the molecular mechanism of action of NPs on HepG2 cells, in the present research we analysed the DNA methylation status of genes related to inflammation and apoptosis, as well as the expression of miRNAs related to these processes in response to AgNPs, AuNPs and SPIONs at low cytotoxic doses.

## 2. Materials and Methods

### 2.1. Nanoparticle Preparation

AgNPs of nominal size 20 nm were purchased from Plasmachem GmbH, Berlin, Germany. AgNPs stock solution was prepared as previously described [39]. In brief, AgNPs (2 mg) were suspended in 800 μL of distilled water and sonicated in an ice water bath with a dose of 4.2 kJ/cm^3^. One hundred microliters of 15% bovine serum albumin and 100 μL of a 10× concentrated phosphate-buffered saline (PBS) were added immediately after sonication. Detailed characteristics of the AgNPs in different culture media, including hydrodynamic size, zeta potential, aggregation rate and stability in cell culture media can be found in our previous papers [36,39,40].

Sodium-citrate-coated AuNPs and PVP-coated magnetite (Fe_3_O_4_) nanoparticles of 20 nm nominal size were purchased from NanoComposix, San Diego, CA, USA. According to the manufacturer’s data, their hydrodynamic diameters were 24 and 40 nm and zeta potentials were −43.6 and −49.7 mV, respectively. AuNPs were diluted in the cell culture medium and used in the experiments without additional processing. SPIONs stock solution (20 mg/mL) was diluted in sterile deionised water to 1 mg/mL and sonicated for 10 min in an ice water bath with a dose of 4.2 kJ/cm^3^. The stability of AuNPs and SPIONs in the cell culture medium can be found in our previous paper [36].

### 2.2. Cell Culture

Human hepatic cell line HepG2 was purchased from the American Type Culture Collection (ATCC, Manassas, VA, USA), and cells were cultured in EMEM medium (ATCC) supplemented with 10% foetal calf serum (Gibco, Thermo Fisher Scientific, Waltham, MA, USA). The cells were incubated in 5% CO_2_ atmosphere at 37 °C. Asynchronous cell cultures in the exponential phase of growth were used in all experiments.

### 2.3. Neutral Red Assay

The neutral red (NR) assay was performed as described previously [41]. Briefly, HepG2 cells were seeded in 96-well microplates (TPP, Trasadingen, Switzerland) at a density of 1.5 × 10^4^ cells/well in 200 µL of culture medium. Twenty-four hours after seeding, cells were treated with nanoparticles (10 µg/mL AgNPs, 10 µg/mL AuNPs, 5 µg/mL SPIONs). After 24 h, the cell culture medium was removed, the cells were washed with 150 µL PBS and incubated for 3 h at 37 °C with 100 µL of the neutral red solution at a final concentration of 40 μg/mL. Next, the NR solution was aspirated, cells were washed with 150 µL of PBS, and 150 µL of an acetic acid–ethanol solution (49% water, 50% ethanol and 1% acetic acid) was added to each well. After 15 min of gentle shaking, the optical density was read at 540 nm in the plate reader spectrophotometer Infinite M200 (Tecan, Männedorf, Switzerland). Four independent experiments in three replicate wells were conducted per experimental point.

### 2.4. Methylation Analysis

For DNA methylation analysis, cells were seeded onto 60-mm cell culture dishes 24 h before treatment. Subsequently, nanoparticle stock suspension (or carrier mix for control plate) was added directly to the cell culture to obtain the final concentration (10 µg/mL AgNPs, 10 µg/mL AuNPs, 5 µg/mL SPIONs). After incubation for 24 h, the cells were washed with PBS, trypsinised and harvested for DNA isolation. Genomic DNA was isolated from the cell pellets using the DNeasy Blood and Tissue kit (Qiagen, Hilden, Germany) using a protocol supplied by the manufacturer. Promoter methylation of genes related to inflammatory response and apoptosis was analysed using EpiTect Methyl II PCR Array Human Inflammatory Response & Autoimmunity Signature Panel and EpiTect Methyl II PCR Array Human Apoptosis Signature Panel (Qiagen, Hilden, Germany). The method is based on detecting remaining DNA input after cleavage with methylation-sensitive and/or methylation-dependent restriction enzymes. The DNA was digested using EpiTect II DNA Methylation Enzyme Kit (Qiagen, Hilden, Germany) following the manufacturer’s protocol. After digestion, quantification of remaining DNA in each enzyme reaction was carried out by real-time PCR using RT^2^ SYBR Green ROX qPCR Mastermix (Qiagen, Hilden, Germany) in the 7500 Real-Time PCR system (Applied Biosystems, Thermo Fisher Scientific, Waltham, MA, USA). The thermal cycler was programmed according to the manufacturer’s instructions, using the following PCR cycling protocol: 1 cycle of 95 °C for 10 min, 3 cycles of 99 °C for 30 s, 72 °C for 1 min; 40 cycles of 97 °C for 15 s, 72 °C for 1 min. The assay provided gene methylation status as a percentage of the methylated and unmethylated fractions of input DNA. Each array included specific control assays for monitoring the cutting efficiencies of methylation-sensitive and methylation-dependent enzymes and ensuring reliable and reproducible results. Data were assessed using the Excel template released by Qiagen, Hilden, Germany Version 2.0, 02/03/2012.

### 2.5. Analysis of miRNA Expression by Real-Time PCR

For miRNA expression experiments, cells were seeded onto 60-mm cell culture dishes 24 h before treatment. Subsequently, nanoparticle stock suspension (or carrier mix for control plate) was added directly to the cell culture to obtain the final concentration (10 µg/mL AgNPs, 10 µg/mL AuNPs, 5 µg/mL SPIONs). After incubation for 6 h, the cells were washed with PBS, trypsinised and harvested for RNA isolation. Total RNA including miRNA was extracted from the cell pellets using a miRNeasy Mini Kit (Qiagen, Hilden, Germany) according to the manufacturer’s protocol. The RNA concentration was measured using a Quantus Fluorometer (Promega, Madison, WI, USA) and the QuantiFluor RNA System (Promega, Madison, WI, USA). Reverse transcription reactions were performed using a miScript II RT Kit (Qiagen, Hilden, Germany). The reaction mix contained 250 ng template RNA, 1× miScript HiSpec Buffer (to specifically convert mature miRNAs into cDNA), 1× miScript Nucleics Mix, RNase-free water and miScript Reverse Transcriptase Mix in a final volume of 20 μL. Reverse transcription mix was incubated for 60 min at 37 °C, followed by 5 min at 95 °C to inactivate the reverse transcriptase. Subsequently, samples were diluted with 200 µL RNase-free water and submitted to real-time PCR analysis using miScript miRNA PCR Array Human Apoptosis (Qiagen, Hilden, Germany), miScript miRNA PCR Array Human Inflammatory Response & Autoimmunity (Qiagen, Hilden, Germany) and miScript SYBR Green PCR Kit (Qiagen, Hilden, Germany). PCR amplification was performed using a 7500 Real-Time PCR System (Applied Biosystems, Thermo Fisher Scientific, Waltham, MA, USA) with an initial 15 min step at 95 °C followed by 40 cycles of 94 °C for 15 s, 55 °C for 30 s and 70 °C for 34 s. Relative miRNA expression was calculated using the ΔΔCt method with SNORD61, SNORD68, SNORD95 and SNORD96A as endogenous controls. Calculations were done using Relative Quantification Software version 3.2.1-PRC-build1 (Thermo Fisher Cloud, Thermo Fisher Scientific, Waltham, MA, USA). Statistical differences were examined by Student’s *t*-test with *p* < 0.05 considered to be statistically significant.

### 2.6. Statistical Evaluation

Except for miRNA expression, statistical analysis of the obtained data was performed using Statistica 7.1 software (StatSoft, Tulsa, OK, USA). The data were expressed as the mean ± standard deviation from at least three independent experiments. Statistical significance was evaluated using Student’s *t*-test. Differences were considered statistically significant when the *p*-value was <0.05.

## 3. Results

### 3.1. Nanoparticles in Selected Concentrations Have a Marginal Effect on Cell Viability

**Figure 1:**
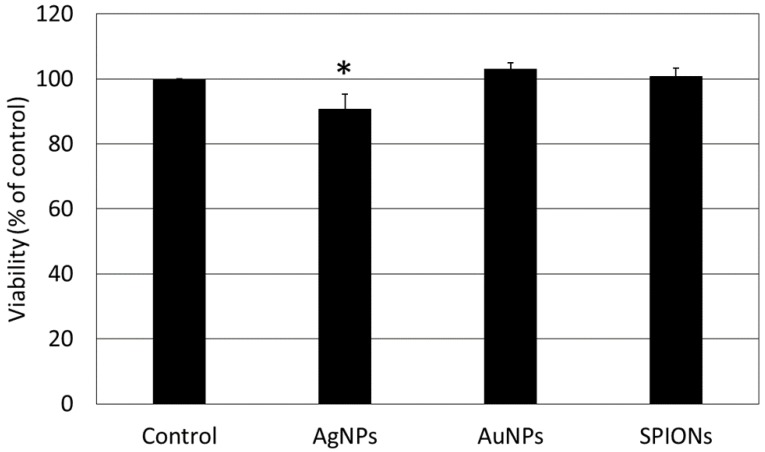
The viability of HepG2 cells measured by the neutral red assay. Cells were treated with silver nanoparticles (AgNPs; 10 µg/mL), gold nanoparticles (AuNPs; 10 µg/mL) and superparamagnetic iron oxide nanoparticles (SPIONs; 5 µg/mL) for 24 h. Data are presented as the mean ± standard deviation from four independent experiments. Asterisk denotes a statistically significant difference versus the control group.

Based on our previous research, for the present study we chose doses of nanoparticles that expressed minimal toxicity in HepG2 cells. The impact of AgNPs, AuNPs and SPIONs on cell viability was confirmed in the neutral red assay. It revealed that selected concentrations of AuNPs and SPIONs had no significant impact on the viability of HepG2 cells after 24 h treatment. Still, a small decrease in HepG2 viability after AgNPs treatment was observed (Figure 1). Internalization of the NPs under study into HepG2 cells was proven using flow cytometry as reported in our previous paper [36].

### 3.2. Nanoparticles Do Not Affect the Methylation Status of Genes Related to Apoptosis and Inflammation

HepG2 cells were treated with NPs for 24 h, and the methylation status of the promoter region of 44 genes related to apoptosis and inflammatory response was compared between treated and control cells using EpiTect Methyl II PCR Arrays. There were no significant differences between treated and control cells in the methylation status of genes under study for any type of nanoparticles. Thirty-one genes were unmethylated, six were methylated, and seven were partially methylated. The detailed results are presented in Table 1.

### 3.3. Nanoparticles Induce Changes in miRNA Expression

**Figure 2:**
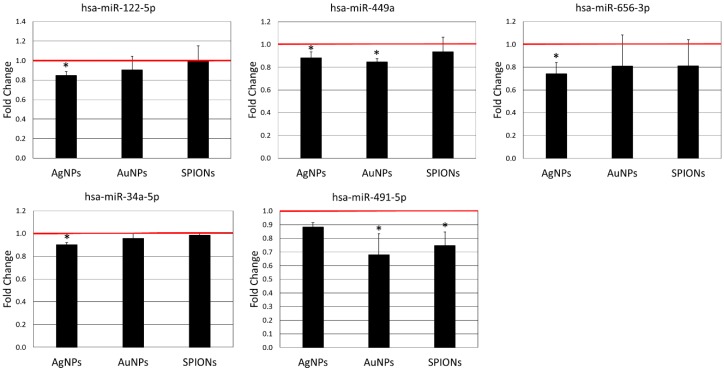
Expression of miRNAs down-regulated by AgNPs (10 µg/mL), AuNPs (10 µg/mL) or SPIONs (5 µg/mL) treatment for 6 h. Data are expressed as the mean ± standard deviation from three independent experiments. The red line shows the expression level in the control group. Asterisks denote a statistically significant difference versus the control group.

**Figure 3:**
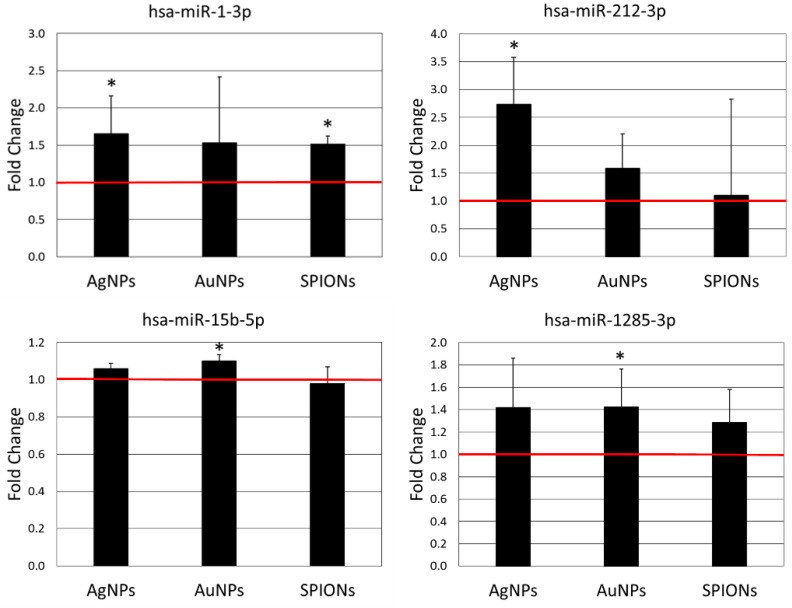
Expression of miRNAs up-regulated by AgNPs (10 µg/mL), AuNPs (10 µg/mL) or SPIONs (5 µg/mL) treatment for 6 h. Data are expressed as the mean ± standard deviation from three independent experiments. The red line shows the expression level in the control group. Asterisks denote a statistically significant difference versus the control group.

In order to check if nanoparticles affect the expression of 131 miRNAs related to inflammatory response and apoptosis, we analysed their expression in HepG2 cells treated with nanoparticles using miScript miRNA PCR Array Human Apoptosis and miScript miRNA PCR Array Human Inflammatory Response & Autoimmunity. We identified nine miRNAs, the expression of which was significantly changed by treatment with nanoparticles. In five cases expression was decreased (Figure 2) and in four cases expression was increased (Figure 3) after treatment with NPs. As could be expected, the different types of nanoparticles did not act in the same way. The highest number of changes was induced by AgNPs (six miRNAs), followed by AuNPs (four miRNAs) and SPIONs (two miRNAs). Interestingly, the overlap between different types of NPs was quite small. Only three miRNAs were induced by more than one type of NPs, that is, miR-499a (expression decreased by AgNPs and AuNPs), miR-491-5p (expression decreased by AuNPs and SPIONs) and miR-1-3p (expression increased by AgNPs and SPIONs). The detailed results of miRNA expression analysis can be found in Supplementary Table S1.

## 4. Discussion

DNA methylation is a covalent chemical modification of cytosines at the C5 position with a methyl group and occurs at CpG dinucleotides clustered into CpG islands. It affects DNA accessibility to the cellular transcriptional machinery and typically inhibits gene expression [42]. In our experimental setup, we did not observe any changes in the promoter methylation of genes related to inflammation and apoptosis in HepG2 cells after treatment with AgNPs, AuNPs and SPIONs (Table 1). To our knowledge, this is the first report on DNA methylation in HepG2 cells following nanoparticles exposure and the first report on the analysis of DNA methylation after treatment with magnetite (Fe_3_O_4_) SPIONs. It has been previously reported that AgNPs induce global DNA methylation in A549 [20] and HT22 cells [21], and a gene-specific methylation in BEAS-2B cells [43] and zebrafish embryos [44]. The only report concerning the impact of AuNPs on DNA methylation shows the induction of methylation of several genes in mouse lungs in vivo [17]. Similarly, the only report regarding SPIONs’ impact on DNA methylation shows that maghemite (γ-Fe_2_O_3_) nanoparticles did not affect global DNA methylation in MCF-7 cells, but affect the methylation of several specific genes [33]. It seems that the impact of nanoparticles on DNA methylation is cell-type-specific and highly dependent on the properties of the nanomaterial. Nevertheless, the present results suggest that gene expression changes observed previously in HepG2 cells after NPs treatment [36] are unlikely to be induced by changes in DNA methylation.

One of the most frequently studied epigenetic mechanisms is miRNA expression. miRNAs are endogenous, short (~22 nucleotides), non-coding RNAs regulating gene expression at post-transcriptional and translational levels via complementary base-pairing with target mRNA. miRNAs play an essential role in a broad range of cellular processes, including proliferation, differentiation, stress response and apoptosis [45]. High complementarity of miRNA and its mRNA target results in mRNA cleavage through an RNA interference mechanism. In contrast, partial complementarity, which is commonly seen in mammalian cells, results in translational inhibition. Single miRNAs can simultaneously regulate multiple targets or even whole biological networks [46].

The effect of AgNPs on miRNA expression has been tested only in vitro so far, with significant miRNA expression changes observed in human dermal fibroblasts [47], Jurkat cells [11] and neural cells [12]. Similarly, the modulation of miRNA expression by AuNPs has been shown in vitro in human dermal fibroblasts [7] and lung fibroblasts [10]. It has also been reported that administration of 100 nm AuNPs to pregnant mice alters miRNA expression in foetal lungs and liver. Interestingly, 40 nm AuNPs did not produce any adverse effects in this experimental system [6]. To our knowledge, the impact of Fe_3_O_4_ SPIONs on miRNA expression has not been studied so far. Li et al. reported that in NIH/3T3 cells Fe_2_O_3_ SPIONs affected the expression of various miRNAs, including an increased expression of miR-34a [9]. Similarly, Huang et al. reported an increase in miR-34a expression in human dermal fibroblasts in response to AuNPs [7]. In our study, miR-34a was slightly downregulated by AgNPs, but was not affected by AuNPs nor Fe_3_O_4_ SPIONs (Figure 2).

Guennewig et al. have shown that miR-34a targets the tumour necrosis factor (TNF) gene and inhibits LPS-induced TNF expression in macrophages [48]. In our previous study, we observed a synergistic effect of AgNPs and TNF on TNF gene expression in HepG2 cells [36], which may be attributed to the suppression of miR-34a expression by AgNPs observed in this study. On the other hand, Wan et al. showed that miR-34a was induced by oxidative stress in a p53-dependent manner in hepatocellular carcinoma [49]. As oxidative stress induction is regarded as a primary mechanism of AgNPs’ action inside a cell [39], increased miR-34a level in the AgNPs-treated cells could be expected. This is in contrast with the decreased miR-34a expression observed in our study. As miR-34a induction in response to oxidative stress is dependent on p53 activation, we can speculate that in our study oxidative stress caused by AgNPs was not sufficient to induce a significant amount of DNA damage to trigger p53 activation.

All of the miRNAs affected by treatment with NPs in the present study, except miR-15b-5p, have previously been shown to inhibit cell proliferation and tumourigenesis via different mechanisms [50,51,52,53,54,55,56,57]. miR-15b-5p was previously reported both as facilitating or suppressing tumorigenesis [58,59]. Modulation of miRNA expression by NPs constitutes a possible mechanism by which NPs can promote or inhibit tumour initiation and/or progression. However, based on the observed changes in miRNA expression we cannot draw a definite conclusion regarding the pro- or anti-tumour nature of the NPs under study. The observed pattern of miRNA expression induced by NPs is ambiguous. All NPs under study both induce and inhibit expression of miRNAs with reported tumour-suppressing activity (Figure 2 and Figure 3).

AgNPs, AuNPs and SPIONs are already used or are planned to be used in numerous applications in biology and medicine as anti-microbial agents, radio-sensitizers, drug carriers and contrast agents. The effects of these NPs on miRNA expression observed in the present work as well as previously reported effects on the gene expression and activity of cellular signalling pathways [34,35,36] do not preclude their usefulness in biological and medical applications. Nevertheless, the observed effects must be taken into consideration during the development of such applications.

Taken together, the results of the present study support the idea of including epigenetic testing during the toxicological assessment of the biological interaction of nanomaterials. For the first time, we have shown that commonly used NPs induce miRNA expression changes in HepG2 cells. We have also shown that the mechanism of action of these NPs on HepG2 cells is not related to the changes in methylation of the promoter region of genes related to inflammation and apoptosis. Further research is needed to fully elucidate the relationship between observed changes in miRNA expression and the effects of NPs observed at the cellular level.

## Figures and Tables

**Table 1:** Methylation analysis of the promoter region of selected genes related to inflammatory response and apoptosis. HepG2 cells were treated with AgNPs (10 µg/mL), AuNPs (10 µg/mL) and SPIONs (5 µg/mL) for 24 h. Data are presented as the mean from three independent experiments. Differences were not statistically significant.

Gene Symbol	% Methylated			
	Control	AgNPs	AuNPs	SPIONs
*APAF1*	0	0	0	0
*ATF2*	0	0	0	0
*BAD*	0	0	0	0
*BAX*	0	0	0	0
*BCL2L11*	100	100	100	100
*BCLAF1*	0	0	0	0
*BID*	0	0	0	0
*BIK*	63	64	58	62
*BIRC2*	0	0	0	0
*BNIP3L*	0	0	0	0
*CASP3*	0	0	0	0
*CASP9*	2	2	3	2
*CCL25*	71	72	68	70
*CIDEB*	100	100	100	100
*CRADD*	0	0	0	0
*CXCL14*	75	71	71	72
*CXCL3*	66	63	66	65
*CXCL5*	76	72	75	75
*CXCL6*	99	99	99	99
*DAPK1*	0	0	0	0
*DFFA*	0	0	0	0
*FADD*	0	0	0	0
*FADD*	0	0	0	0
*GADD45A*	0	0	0	0
*GATA3*	0	0	0	0
*HRK*	0	0	0	0
*IL10RA*	78	78	72	75
*IL12A*	0	0	0	0
*IL12B*	0	0	0	1
*IL13*	96	99	98	93
*IL13RA1*	1	1	1	6
*IL15*	0	0	0	0
*IL17C*	100	100	100	100
*IL17RA*	66	63	66	64
*IL4R*	0	0	0	1
*IL6R*	0	0	0	0
*IL6ST*	0	0	0	0
*IL7*	0	0	0	0
*INHA*	0	0	0	0
*LTBR*	0	0	0	0
*TNFRSF21*	0	0	0	0
*TNFRSF25*	100	100	100	100
*TP53*	0	0	0	0
*TYK2*	0	0	0	1

## Supplementary Materials

The following are available online at https://www.mdpi.com/1996-1944/12/7/1038/s1. Table S1: The detailed results of miRNA expression analysis.
