# Supplementary material for: Silver, Gold, and Iron Oxide Nanoparticles Alter miRNA Expression but Do Not Affect DNA Methylation in HepG2 Cells
Source: Materials (Basel). 2019 Mar 29;12(7):1038. doi: 10.3390/ma12071038 (PMC6479689; doi:10.3390/ma12071038)
Supplement: Supplementary file 1 [file materials-12-01038-s001.pdf]

Supplementary Table 1. Analysis of miRNA expression in HepG2 cells treated with AgNPs (10 µg/mL), AuNPs (10 µg/mL), and SPIONs (5 µg/mL) for 24 h. Mean fold changes from three independent experiments. *p* values <0.05 are marked in red.

Target Name	AgNPs		AuNPs		SPIONs	
	Fold Change	*p* value (t-test)	Fold Change	*p* value (t-test)	Fold Change	*p* value (t-test)
hsa-let-7a-5p	1.023	0.679	1.032	0.263	0.986	0.857
hsa-let-7b-5p	0.837	0.589	0.853	0.249	1.376	0.143
hsa-let-7c-5p	1.047	0.833	1.038	0.625	1.021	0.803
hsa-let-7d-5p	1.123	0.190	1.170	0.282	0.967	0.794
hsa-let-7e-5p	0.936	0.409	0.963	0.529	0.856	0.317
hsa-let-7f-5p	0.987	0.732	1.020	0.715	0.951	0.712
hsa-let-7g-5p	0.988	0.870	1.052	0.456	0.981	0.875
hsa-let-7i-5p	0.942	0.641	0.955	0.545	0.825	0.094
hsa-miR-101-3p	0.979	0.824	0.943	0.539	0.995	0.957
hsa-miR-106b-5p	0.952	0.598	0.964	0.656	0.963	0.619
hsa-miR-122-5p	0.847	0.021	0.903	0.351	0.991	0.922
hsa-miR-125a-5p	0.992	0.955	1.037	0.840	0.970	0.853
hsa-miR-125b-5p	0.941	0.722	1.053	0.687	0.883	0.588
hsa-miR-128-3p	0.950	0.564	0.998	0.975	0.943	0.368
hsa-miR-1285-3p	1.418	0.152	1.424	0.048	1.284	0.166
hsa-miR-130a-3p	0.878	0.417	1.031	0.893	1.077	0.578
hsa-miR-130b-3p	0.846	0.172	0.953	0.648	0.933	0.525
hsa-miR-1324	0.866	0.506	0.832	0.446	1.604	0.416
hsa-miR-133a-3p	0.619	0.565	0.850	0.828	0.751	0.725
hsa-miR-133b	0.383	0.325	1.165	0.899	0.613	0.579
hsa-miR-134-5p	0.851	0.540	0.919	0.839	0.923	0.758
hsa-miR-1-3p	1.650	0.043	1.534	0.240	1.511	0.012
hsa-miR-141-3p	1.070	0.672	0.977	0.913	0.887	0.500
hsa-miR-143-3p	1.025	0.663	1.164	0.182	1.063	0.485
hsa-miR-144-3p	2.004	0.244	2.007	0.311	2.133	0.342
hsa-miR-145-5p	0.930	0.294	1.006	0.912	0.958	0.270
hsa-miR-146a-5p	0.968	0.224	1.029	0.722	0.955	0.178
hsa-miR-149-3p	0.939	0.741	0.936	0.717	0.996	0.981
hsa-miR-153-3p	0.952	0.584	0.998	0.983	0.829	0.109
hsa-miR-15a-5p	0.926	0.576	0.993	0.945	1.030	0.753
hsa-miR-15b-5p	1.058	0.155	1.100	0.047	0.980	0.745
hsa-miR-16-5p	0.959	0.516	1.003	0.950	0.954	0.455
hsa-miR-17-5p	0.979	0.752	1.016	0.775	0.953	0.546
hsa-miR-181a-5p	0.910	0.664	1.029	0.760	1.127	0.267
hsa-miR-181b-5p	1.076	0.528	1.009	0.936	0.962	0.712
hsa-miR-181c-5p	0.916	0.364	1.025	0.819	0.992	0.956
hsa-miR-181d-5p	1.153	0.410	1.050	0.775	0.971	0.841
hsa-miR-183-5p	1.104	0.243	1.060	0.275	0.941	0.474
hsa-miR-185-5p	0.944	0.238	1.012	0.766	1.012	0.860

hsa-miR-186-3p	1.196	0.894	0.639	0.702	3.105	0.333
hsa-miR-186-5p	0.871	0.263	0.959	0.579	0.894	0.192
hsa-miR-192-5p	0.959	0.670	1.036	0.548	0.998	0.981
hsa-miR-193a-5p	0.937	0.783	1.097	0.547	0.935	0.369
hsa-miR-193b-3p	0.949	0.594	0.883	0.209	0.996	0.966
hsa-miR-194-5p	0.938	0.354	1.039	0.415	0.949	0.482
hsa-miR-195-5p	1.007	0.908	1.027	0.712	0.970	0.687
hsa-miR-19a-3p	0.921	0.406	0.929	0.436	0.897	0.229
hsa-miR-19b-3p	0.914	0.258	0.924	0.333	0.905	0.216
hsa-miR-200c-3p	1.013	0.792	1.064	0.267	0.931	0.468
hsa-miR-202-3p	1.989	0.374	1.913	0.419	1.514	0.604
hsa-miR-203a-3p	7.356	0.106	0.910	0.929	1.382	0.764
hsa-miR-204-5p	1.239	0.260	1.313	0.122	1.045	0.733
hsa-miR-205-5p	3.290	0.255	3.510	0.234	4.039	0.199
hsa-miR-206	0.640	0.471	0.735	0.608	0.584	0.398
hsa-miR-20a-5p	0.962	0.620	0.991	0.895	0.949	0.462
hsa-miR-20b-5p	0.923	0.465	0.958	0.655	0.910	0.363
hsa-miR-210-3p	0.892	0.367	0.959	0.692	0.978	0.849
hsa-miR-211-5p	0.652	0.424	0.680	0.466	1.084	0.926
hsa-miR-212-3p	2.735	0.041	1.588	0.233	1.100	0.885
hsa-miR-214-3p	1.818	0.059	1.296	0.672	1.484	0.329
hsa-miR-21-5p	1.035	0.710	1.013	0.900	0.961	0.684
hsa-miR-218-5p	0.826	0.390	1.114	0.426	0.851	0.274
hsa-miR-221-3p	0.992	0.886	1.020	0.741	0.969	0.549
hsa-miR-222-3p	0.966	0.602	0.966	0.699	0.939	0.455
hsa-miR-23a-3p	1.161	0.464	1.041	0.835	0.956	0.815
hsa-miR-23b-3p	0.964	0.479	0.984	0.800	0.891	0.191
hsa-miR-24-3p	1.029	0.665	1.031	0.638	0.970	0.624
hsa-miR-25-3p	1.007	0.857	1.057	0.332	0.935	0.573
hsa-miR-26a-5p	0.989	0.593	1.044	0.047	0.950	0.480
hsa-miR-26b-5p	1.054	0.160	1.102	0.123	0.952	0.695
hsa-miR-27a-3p	1.090	0.617	0.994	0.972	0.956	0.790
hsa-miR-29a-3p	1.000	0.998	1.021	0.888	1.021	0.870
hsa-miR-29b-3p	1.065	0.484	0.985	0.729	1.027	0.787
hsa-miR-29c-3p	1.030	0.823	1.048	0.739	1.042	0.752
hsa-miR-300	0.793	0.705	1.769	0.437	0.511	0.304
hsa-miR-301a-3p	0.971	0.541	0.971	0.482	0.967	0.425
hsa-miR-301b-3p	1.009	0.888	0.958	0.534	0.943	0.358
hsa-miR-302a-3p	0.524	0.463	2.037	0.201	0.847	0.712
hsa-miR-302b-3p	0.653	0.577	0.627	0.547	0.573	0.479
hsa-miR-302c-3p	5.903	0.112	1.773	0.471	1.016	0.981
hsa-miR-30a-5p	0.991	0.810	0.999	0.969	0.954	0.499
hsa-miR-30b-5p	0.973	0.711	0.977	0.596	0.932	0.424
hsa-miR-30c-5p	0.961	0.675	1.049	0.542	0.962	0.725
hsa-miR-30d-5p	1.010	0.786	1.007	0.803	0.950	0.505

hsa-miR-30e-5p	1.006	0.941	0.989	0.863	0.973	0.717
hsa-miR-31-5p	1.558	0.398	0.658	0.313	1.122	0.784
hsa-miR-32-5p	0.974	0.923	1.084	0.732	1.145	0.569
hsa-miR-338-3p	0.955	0.825	0.958	0.819	0.992	0.968
hsa-miR-340-5p	0.975	0.744	1.031	0.619	0.887	0.190
hsa-miR-34a-5p	0.901	0.034	0.959	0.361	0.984	0.667
hsa-miR-34c-5p	1.621	0.611	2.481	0.294	2.201	0.286
hsa-miR-365a-3p	1.047	0.040	0.993	0.758	0.919	0.249
hsa-miR-372-3p	0.777	0.703	1.785	0.138	1.193	0.491
hsa-miR-373-3p	1.623	0.487	0.570	0.615	2.020	0.277
hsa-miR-374a-5p	1.054	0.468	1.104	0.251	0.920	0.408
hsa-miR-378a-3p	0.860	0.074	0.964	0.682	0.907	0.173
hsa-miR-381-3p	1.905	0.061	1.863	0.240	0.821	0.782
hsa-miR-409-3p	0.652	0.378	0.689	0.613	0.782	0.557
hsa-miR-410-3p	0.916	0.811	0.716	0.441	0.630	0.185
hsa-miR-424-5p	1.627	0.652	0.893	0.899	2.457	0.314
hsa-miR-449a	0.882	0.049	0.846	0.016	0.937	0.485
hsa-miR-449b-5p	0.732	0.219	1.245	0.415	0.392	0.192
hsa-miR-451a	0.613	0.139	0.959	0.859	1.092	0.775
hsa-miR-454-3p	0.946	0.284	0.964	0.557	0.899	0.256
hsa-miR-491-5p	0.881	0.118	0.680	0.040	0.746	0.035
hsa-miR-497-5p	0.919	0.539	0.784	0.117	0.649	0.067
hsa-miR-511-5p	0.701	0.606	0.686	0.430	0.980	0.971
hsa-miR-512-5p	0.577	0.571	0.888	0.931	3.841	0.214
hsa-miR-513b-5p	1.442	0.294	1.273	0.496	1.592	0.437
hsa-miR-519c-3p	1.254	0.796	0.917	0.896	0.510	0.304
hsa-miR-519d-3p	0.392	0.268	0.283	0.156	0.296	0.170
hsa-miR-520d-3p	0.691	0.683	0.800	0.541	0.963	0.930
hsa-miR-520e	4.069	0.247	2.041	0.364	2.425	0.254
hsa-miR-524-5p	0.721	0.617	1.073	0.938	2.584	0.194
hsa-miR-542-3p	0.666	0.551	1.857	0.281	1.209	0.811
hsa-miR-543	1.739	0.552	0.877	0.866	0.954	0.844
hsa-miR-545-3p	1.396	0.130	0.754	0.259	1.293	0.176
hsa-miR-548c-3p	0.486	0.199	0.600	0.501	0.871	0.722
hsa-miR-548d-3p	0.774	0.231	0.989	0.904	0.862	0.185
hsa-miR-548e-3p	0.856	0.427	0.806	0.286	0.843	0.440
hsa-miR-590-5p	0.988	0.890	0.911	0.375	0.966	0.676
hsa-miR-607	1.197	0.514	1.149	0.626	1.951	0.409
hsa-miR-655-3p	1.116	0.899	0.713	0.550	0.526	0.292
hsa-miR-656-3p	0.742	0.041	0.810	0.340	0.811	0.286
hsa-miR-708-5p	2.784	0.375	3.136	0.312	4.198	0.226
hsa-miR-7-5p	1.070	0.283	0.999	0.989	0.965	0.765
hsa-miR-875-3p	1.569	0.266	2.062	0.313	0.865	0.791
hsa-miR-92a-3p	1.003	0.926	1.038	0.333	0.973	0.566
hsa-miR-93-5p	0.973	0.645	1.006	0.927	0.973	0.357

hsa-miR-9-5p	3.979	0.252	1.196	0.859	1.166	0.878
hsa-miR-98-5p	0.911	0.405	1.056	0.613	1.033	0.851
